# A cell-based assay for detection of anti-fibrillarin autoantibodies with performance equivalent to immunoprecipitation

**DOI:** 10.3389/fimmu.2022.1011110

**Published:** 2022-09-26

**Authors:** Gerson Dierley Keppeke, Minoru Satoh, Cristiane Kayser, Pedro Matos, Tomoko Hasegawa, Shin Tanaka, Larissa Diogenes, Rogerio Quintiliano Amaral, Silvia Helena Rodrigues, Luis Eduardo Coelho Andrade

**Affiliations:** ^1^ Rheumatology Division, Department of Medicine, Federal University of Sao Paulo, Sao Paulo, Brazil; ^2^ Department of Clinical Nursing, School of Health Sciences, University of Occupational and Environmental Health, Kitakyushu, Japan; ^3^ Department of Medicine, Kitakyushu Yahata-Higashi Hospital, Kitakyushu, Japan; ^4^ Department of Human, Information, and Science, School of Health Sciences, University of Occupational and Environmental Health, Kitakyushu, Japan; ^5^ Immunology Division, Fleury Laboratory, Sao Paulo, Brazil

**Keywords:** anti-fibrillarin, antinuclear antibodies, autoantibody (ies), biomarkers, cell-based assay, systemic sclerosis

## Abstract

Anti-fibrillarin autoantibodies are useful for the diagnosis and prognosis of systemic sclerosis (SSc). Anti-fibrillarin produces a clumpy nucleolar pattern in indirect immunofluorescence assay on HEp-2 cells (HEp-2 IFA). Here we develop and validate a reliable cell-based anti-fibrillarin assay (Fibrillarin/CBA) for use in clinical diagnostic laboratories. A TransMembrane Signal was fused to the human fibrillarin gene (TMS-fibrillarin). HEp-2 cells overexpressing transgenic TMS-fibrillarin at the cytoplasmic membrane were used as IFA substrate in the Fibrillarin/CBA. Sixty-two serum samples with nucleolar pattern in the HEp-2 IFA (41 clumpy; 21 homogeneous/punctate) were tested for anti-fibrillarin using Fibrillarin/CBA, immunoprecipitation (IP), line-blot and ELISA. In addition, samples from 106 SSc-patients were evaluated with Fibrillarin/CBA and the results were correlated with disease phenotypes. Thirty-eight of 41 samples with the clumpy nucleolar pattern (92.7%) were positive in the Fibrillarin/CBA, while all 21 samples with other nucleolar patterns were negative. Fibrillarin/CBA results agreed 100% with IP results. Among the 38 Fibrillarin/CBA-positive samples, only 15 (39.5%) and 11 (29%) were positive for anti-fibrillarin in line-blot and ELISA, respectively. Higher frequency of diffuse cutaneous SSc (dcSSc) phenotype (72.7% vs 36.8%; p=0.022), cardiac involvement (36.4% vs 6.5%; p=0.001) and scleroderma renal crisis (18.2% vs 3.3% p = 0.028) was observed in SSc patients with positive compared to negative Fibrillarin/CBA result. Performance of Fibrillarin/CBA in the detection of anti-fibrillarin autoantibodies was comparable to the gold standard IP. Positive Fibrillarin/CBA results correlated with disease phenotypes known to be associated with anti-fibrillarin autoantibodies, underscoring the clinical validation of this novel assay.

## Highlights

•Anti-fibrillarin antibodies correlate with a more severe disease phenotype in SSc patients.
•Immunoprecipitation is the gold standard assay for anti-fibrillarin antibodies.
•By relocating transgenic fibrillarin to the cell membrane, we developed the Fibrillarin/CBA, a new cell-based assay (CBA) for anti-fibrillarin.
•Positive/negative results in the Fibrillarin/CBA agreed 100% with immunoprecipitation results.
•The Fibrillarin/CBA showed higher sensitivity than commercially available solid-phase assays.

## Introduction

Systemic sclerosis (SSc) is a chronic heterogeneous systemic autoimmune disease characterized by microvascular dysfunction, activation of the immune system, and cutaneous and visceral fibrosis. SSc has the highest morbidity and mortality rates among immune-mediated rheumatic diseases (1). However, the clinical manifestations and the clinical course of the disease are highly variable. The heterogeneity of the disease may be represented by the subset classification of SSc, i.e., the limited (lcSSc) and diffuse cutaneous subsets (dcSSc). Patients with dcSSc have rapidly diffuse cutaneous thickening, higher frequency of internal organ involvement and worse prognosis. In contrast, patients with lcSSc typically have restricted skin thickening distribution, less severe organ involvement and a better survival (2).

Serum autoantibodies can be detected in over 90% of SSc patients and are very useful for the early diagnosis of SSc and for the identification of certain SSc disease phenotypes. Several of these autoantibodies are highly specific for SSc and help in predicting clinical complications and the prognosis of SSc patients (3, 4). For example, anti-topoisomerase I antibodies are associated with dcSSc, digital ulcers, cardiomyopathy, high skin score, higher risk of severe interstitial lung disease (ILD) (5) and underlying malignancies (6), resulting in a more severe phenotype and increased mortality (4, 7–9). Anti-RNA polymerase III (RNAP III) antibodies are associated with the dcSSc subset, high risk of severe, rapidly progressing cutaneous thickening, higher risk for gastric antral vascular ectasia (10, 11), and scleroderma renal crisis (10, 12), as well as a higher risk of development of malignancy, especially within the first years of disease onset (6, 10). In contrast, anti-centromere antibodies are associated with lcSSc, long-standing Raynaud’s phenomenon, calcinosis, a higher risk of developing pulmonary arterial hypertension (PAH) (13), and better survival rates as compared with patients with anti-topo I and anti-RNAP III antibodies (7).

Anti-U3-RNP/fibrillarin antibodies recognize the U3-ribonucleoprotein (U3-RNP), a nucleolar complex involved in pre-rRNA processing. Fibrillarin is a S-adenosyl-L-methionine-dependent methyltransferase responsible for the methylation of ribose moieties in pre-rRNA (14). It contains 321 amino acids (~34kDa), an N-terminal repetitive domain rich in glycine and arginine residues, and associates with proteins such as NOP56/58, NHP2L1, Mpp10, Imp3/4, among others, to assemble the box C/D RNP complex in the dense fibrillar component of the nucleolus (15, 16). Anti-fibrillarin autoantibodies occurs in up to 10% of SSc patients, especially in African Americans (17, 18). Although the number of studies is small, anti-fibrillarin has been associated with dcSSc and multi-organ involvement (9, 19), with high risk of cardiac involvement, ILD, PAH, renal crisis, small bowel and muscle involvement. In general, anti-fibrillarin antibodies indicate worse prognosis in SSc patients (9, 17).

The indirect immunofluorescence assay on HEp-2 cells (HEp-2 IFA) is the most commonly used method for screening for autoantibodies in systemic autoimmune diseases, including SSc. HEp-2 IFA reveals the pattern of immunofluorescent labeling, a key piece of information indicating the possible location of the autoantigens recognized by autoantibodies in the sample. The International Consensus on ANA Patterns (ICAP) <www.anapatterns.org> has classified 30 different HEp-2 IFA patterns (20), including the nucleolar pattern. Although many will report any nucleolar staining as a “nucleolar pattern”, in a more detailed analysis one can distinguish three different “sub-patterns”: homogeneous nucleolar (AC-8), clumpy nucleolar (AC-9) and punctate nucleolar (AC-10). The nucleolar pattern, usually at high titer, is observed in up to a third of SSc patients (21), although in most studies the proportion of this pattern is around 20% (22–25). A comprehensive review on the topic can be found elsewhere (26). About 30% of samples from patients with SSc with a nucleolar HEp-2 IFA pattern may have anti-fibrillarin (U3-RNP) antibodies, which typically yield a clumpy nucleolar pattern (AC-9) (4, 26). However, the correct classification of the clumpy nucleolar pattern can be challenging as the interpretation is subjective, depends on qualified personnel, and may vary depending on the source of the HEp-2 slides (27). In addition, it is possible that not all clumpy nucleolar patterns are caused by anti-fibrillarin antibodies. Therefore, when a sample presents a nucleolar pattern in the HEp-2 IFA test, and especially if the pattern is indicative of AC-9, additional tests should be performed to confirm the presence of anti-fibrillarin or other autoantibodies that could yield a nucleolar pattern (4).

Commercial kits for detection of anti-fibrillarin antibodies are available. These are based on solid phase immunoassays, such as western-blot, dot-blot, line-blot, fluorescence enzyme immunoassay, ELISA, particle-based multi-analyte technology, among others (28, 29). However, most of these kits are not approved for clinical or diagnostic use. In addition, they can yield inconsistent results with poor sensitivity, possibly because the native conformation of the fibrillarin antigen is not well preserved in these assays, resulting in less efficient antibody binding. The gold standard method for detection of anti-fibrillarin antibodies is immunoprecipitation (IP), an assay in which the native conformation of the fibrillarin antigen is more likely to be preserved (26). Unfortunately, IP is a labor-intensive assay that requires highly skilled analysts and the use of radioactive materials; thus, this method is not widely available in routine clinical laboratories.

In this study, we established an indirect immunofluorescence Cell-Based Assay (CBA) to detect anti-fibrillarin antibodies and compared the performance of this new assay against standard IP and commercial solid phase immunoassays for detection of anti-fibrillarin autoantibody. In addition, we surveyed a cohort of SSc patients with the novel anti-fibrillarin CBA test and confirmed previously observed associations of anti-fibrillarin antibodies and specific SSc phenotypes.

## Materials and methods

### Patient samples

Sixty-two samples with nucleolar pattern at titer ≥1/320 in the HEp-2 IFA test were used for the analytical validation of the Fibrillarin/CBA. These samples were sequentially selected from the routine HEp-2 IFA operation at the Rheumatology Division laboratory, Federal University of Sao Paulo, and at the Fleury clinical laboratory. No identification data or clinical information was made available for these samples. Considering that the samples were used exclusively for immunoassays related to the original physician’s request and no identification or clinical information was used, the Ethics Committee waived the need for informed consent for these 62 samples. For the clinical validation of the Fibrillarin/CBA we tested an additional set of samples from a cohort of 106 patients meeting the American College of Rheumatology/European League Against Rheumatism (ACR/EULAR) 2013 classification criteria for SSc (30), thus determining the frequency and clinical associations of anti-fibrillarin antibodies assayed by this novel methodology. The patients were consecutively recruited from the Rheumatology outpatient clinic at the Federal University of Sao Paulo; their electronic medical records thoroughly reviewed by rheumatologists with expertise in SSc (C.K. and P.M.). In compliance with the Helsinki Declaration, the patients signed an informed consent form to participate in the study, and the research was approved by local Ethics Committee at the Federal University of Sao Paulo.

Clinical and demographic characteristics of the 106 SSc patients, including age, gender, disease subtype (limited or diffuse cutaneous SSc), and disease duration (defined as the time between the first non-Raynaud’s symptom and the last available evaluation), were obtained from medical records as previously described (31).

### Plasmid cloning

Total RNA was extracted from human peripheral blood mononuclear cells (PBMC) using TRIzol (Invitrogen, USA). PBMC was isolated from fresh human blood by density gradient (Ficoll-Paque PLUS 1.077 g/mL, Cytiva, USA). The RNA was converted to complementary DNA (cDNA) with the First Strand cDNA Synthesis Kit (E6300, NEB, USA). From the cDNA, the coding region of fibrillarin gene *FBL* (NCBI Reference Sequence: NM_001436.4) was amplified with the following primers (Forward: ATGAAGCCAGGATTCAGTCCC; Reverse: GTTCTTCACCTTGGGGGGTG), using the Phusion Flash High-Fidelity PCR Master Mix (F548, Thermo Scientific, EUA). Size and sequence were confirmed by agarose gel and Sanger sequencing, respectively.

The TransMembrane Signal (TMS), a proprietary 65-amino acids sequence designed by the study authors (G.D.K. and L.E.C.A.), was added to the N-terminus of the fibrillarin gene to localize the transgenic gene product to the cell membrane (**Supplementary Figure S2**). The cDNA for the TMS fragment was synthesized by TsingKe Biological Technology (China). At the C-terminus of the fibrillarin gene, an orange fluorescent protein (OFP) gene plus a fused myc tag was added, interleaved from *FBL* by a ribosome skipping P2A sequence. The whole cDNA construct was inserted into the linearized vector pCMV3 (Sino Biological, China). For all cloning steps, the Gibson assembly system was used (Kit NEBuilder HiFi DNA Assembly Master Mix [E2621, NEB, USA]). The final plasmid configuration was: pCMV3_TMS-fibrillarin_P2A_OFP-myc.

### Cell transfection and slide preparation

HEp-2 cells were grown to confluence with culture medium DMEM containing 10% fetal bovine serum. The plasmid was transfected with Lipofectamine 3000 Transfection Reagent (L3000008, Invitrogen, USA), diluted in Opti-MEM I Reduced Serum Medium (31985-070, Gibco, USA), following the manufacturer’s protocol. After transfection, cells were seeded in 10-well hydrophobic coated slides (each well with 6-mm diameter) for overnight adherence. After 24h, slides containing the transfected HEp-2 cells were fixed with methanol for 5 min and acetone for 2 min, both cooled to -20°C. After air drying, slides were sealed, packed and stored at -20°C until use (within three months) in indirect immunofluorescence assay (IFA).

### CBA IFA reaction

The IFA protocol is similar to a traditional HEp-2 IFA reaction except for the addition of the anti-tag antibody, performed as previously described (32). Briefly, human sera were diluted 1/80 in PBS containing a mouse anti-Myc monoclonal antibody 9E10 (sc-40, Santa Cruz Biotech, USA), diluted 1/200. Slides containing transfected cells were incubated with this dual antibody mix for 30 min at 37°C in a wet chamber and thereafter washed three times for 5 min with 0.1% Tween in PBS (PBS-T). Next, two secondary antibodies diluted 1/500 in PBS were incubated simultaneously with the cells at 37°C for 30 min in the dark in a wet chamber: anti-human IgG conjugated to Alexa Fluor 488 (A-11013, Invitrogen, USA) and anti-mouse IgG conjugated to Cy3 (715-165-151, Jackson ImmunoResearch, USA). Thereafter slides were washed three times for 5 min with PBS-T, assembled with Vectashield containing DAPI (Vector Labs, USA), and covered with coverslips before analysis in a fluorescence microscope with 200x or 400x magnification (Axio Imager.M2, Carl Zeiss, Germany). For some reactions a mouse monoclonal anti-fibrillarin antibody (clone 72B9 (33)), kindly donated by Professor K. Michael Pollard (The Scripps Research Institute, CA) was used at a dilution of 1/100 to label fibrillarin (**Supplementary Figure S1A**). For human sera that gave inconclusive results in the CBA test when diluted at 1/80, CBA was repeated with serial dilutions, up to 1/1280.

### Sample testing

Anti-cell antibody titer and the HEp-2 IFA pattern, including the nucleolar patterns (homogeneous nucleolar AC-8, clumpy nucleolar AC-9 or punctate nucleolar AC-10) were determined using traditional HEp-2 cell IFA slides (Euroimmun, Germany) starting at 1/80 dilution with sequential double dilutions up to end titer.

All samples were tested for anti-fibrillarin antibody using the CBA test, as described above, and other standard methods for determination of anti-fibrillarin antibodies, as follows. Immunoprecipitation was performed as previously described (34–36), using [35S]-methionine-radiolabeled K562 cell (human erythroleukemia) lysates (**Supplementary Figure S5**).

Line blot assay was performed using the Euroline Systemic Sclerosis (Nucleoli) profile kit (Cat# DL 1532-6401 G, Euroimmun, Germany), following the manufacturer’s protocol. Although this kit can determine reactivity to other antigens, for this study we only considered reactivity to the recombinant fibrillarin (**Supplementary Figure S6**). ELISA analysis was performed using a qualitative anti-fibrillarin antibody ELISA kit (Cat# MBS701068, MyBioSource, USA), following the manufacturer’s protocol.

### Statistical analysis

Classificatory variables (proportions) were compared with two-tailed Chi squared test. Quantitative and semi-quantitative parameters were tested for Gaussian distribution with “D’Agostino and Pearson normality test”. According to the distribution pattern, they were analyzed by the Mann-Whitney or Student t-test. Error bars indicating average and standard deviation (SD) are shown. P values were considered significant when below 0.05. All analyses were performed using the software GraphPad Prism 7.0 for Windows.

## Results

A TransMembrane Signal (TMS) was fused to the N’ terminus of the human fibrillarin gene to localize the transgenic fibrillarin to the cytoplasmic membrane. HEp-2 cells transfected with the plasmid pCMV_TMS-fibrillarin_P2A_OFP-myc were used as substrate for IFA in the CBA. IFA with an anti-fibrillarin mouse monoclonal antibody confirmed that TMS-fibrillarin was successfully positioned at the cell membrane (**Supplementary Figure S1A**). To confirm that the TMS localized the antigen at the cell’s surface, the signal was fused to GFP, overexpressed in HeLa cells and labeled by an anti-GFP antibody, without membrane permeabilization (arrows in **Supplementary Figure S2**). Live cells expressing TMS-fibrillarin could be readily identified by the Orange Fluorescent Protein (OFP) from the construct (data not shown). However, since fixing with methanol/acetone affects OFP fluorescence, labeling of the transfected cells was enhanced using an anti-myc tag monoclonal antibody (**Supplementary Figure S1B**). Quantification of cells expressing TMS-fibrillarin (by myc labeling) 24 hours after transfection (n=727 cells counted) showed transfection efficiency at 18.7% ( ± 3.3%), indicating that about one fifth of the cells express the TMS-fibrillarin.

**Figure 1:**
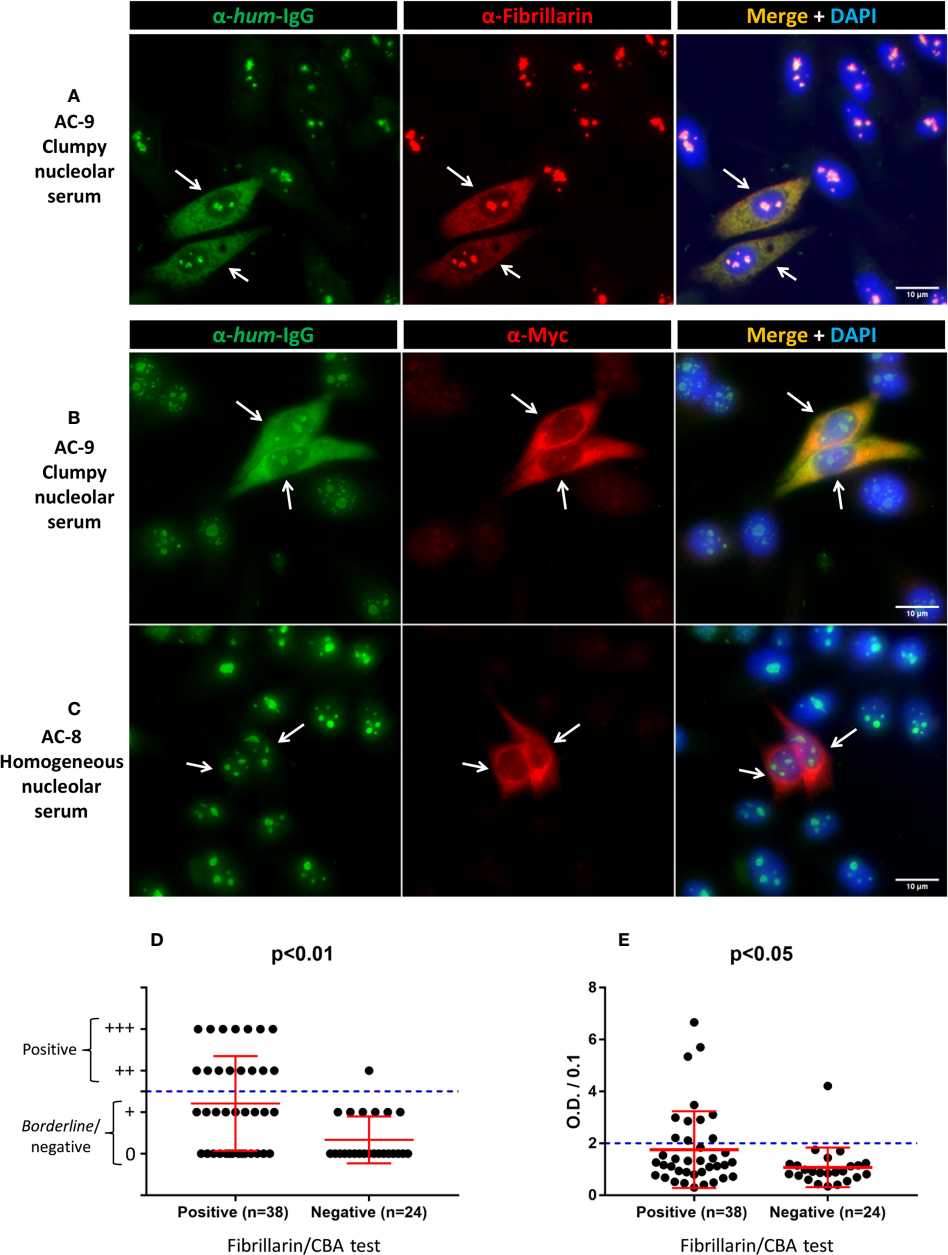
Labeling of TMS-fibrillarin by serum with clumpy nucleolar pattern in HEp-2 IFA. HEp-2 cells transfected with the pCMV_TMS-fibrillarin_P2A_OFP-myc plasmid were labeled with human serum (1/80 dilution) plus mouse monoclonal antibodies, then counterstained with DAPI to visualize DNA. **(A)** Double IIF using human serum with clumpy nucleolar pattern (AC-9) plus mouse anti-fibrillarin. **(B)** Double IIF using a second human serum with clumpy nucleolar pattern (AC-9) plus mouse anti-myc. **(C)** Double IIF using human serum with homogeneous nucleolar pattern (AC-8) plus mouse anti-myc. Arrows indicate TMS-fibrillarin expressing cells. Scale bar = 10µm. **(D, E)** Fibrillarin/CBA test and other methods in 62 samples. **(D)** Line blot assay for anti-fibrillarin antibody. The plus (+) symbols in the *y*-axis indicate the intensity of staining of the fibrillarin line; ≥2 plus (++) was considered positive. **(E)** ELISA test for anti-fibrillarin antibody. Optical Density (O.D.) was divided by 0.1 and values >2 were considered positive as recommended by the manufacturer. **(D, E)** Error bars indicate mean ± SD.

In the initial evaluation of the IIF cell-based assay (Fibrillarin/CBA test), we showed that two human sera that present a clumpy nucleolar pattern (AC-9) in the traditional HEp-2 IFA, but not a serum that presents a homogeneous nucleolar (AC-8) pattern, stained the nucleoli and the cytoplasmic membrane of cells expressing TMS-fibrillarin (**Figure 1**). For analytical validation, we selected 62 samples with strong nucleolar pattern (titer ≥1/320) on the HEp-2 IFA from samples collected during routine laboratory testing. Antibody titer and the nucleolar pattern (homogeneous nucleolar AC-8, clumpy nucleolar AC-9, or punctate nucleolar AC-10) were confirmed with commercial HEp-2 IFA slides. Classification of the nucleolar patterns followed the ICAP recommendations. Representative images of samples with the different nucleolar patterns are shown in **Supplementary Figures S3A–E**.

We intentionally enriched this collection with samples yielding the clumpy nucleolar (AC-9) pattern. Of the 62 samples, 41 (66%) were classified as clumpy nucleolar (AC-9) and 21 (34%) as other nucleolar patterns (20 as AC-8 and 1 as AC-10). Thirty-eight of the 41 AC-9/clumpy nucleolar samples (92.7%) were positive in the Fibrillarin/CBA test, while all 21 samples with other nucleolar patterns were negative (**Table 1**). Representative images of positive and negative samples are shown in **Figure 1** and **Supplementary Figure S4**. Samples that were positive in the Fibrillarin/CBA showed higher HEp-2 IFA titer than samples that were negative (**Supplementary Figure S3F**).

**Table 1:** Performance of the Fibrillarin/CBA test and other methods for determination of anti-fibrillarin antibodies.

		Fibrillarin/CBA test		*P*
		Positive (n = 38) (61.3%)	Negative (n = 24) (38.7%)	
Samples (n = 62)				
HEp-2 IFA nucleolar pattern	Clumpy nucleolar (AC-9) (n=41; 66%)	38 (92.7%)	3 (7.3%)	–
	Homogeneous or punctuate nucleolar (AC-8 and AC-10) (n=21; 34%)	0 (0%)	21 (100%)
Immunoprecipitation fibrillarin reactivity (n=56)	Positive (n=35; 62.5%)	35 (100%)	0 (0%)	p < 0.001
	Negative (n=21; 37.5%)	0 (0%)	21 (100%)
Line blot for anti-fibrillarin (Euroline) (n=62)	Positive (++, +++) (n=16; 25.8%)	15 (93.7%)	1 (6.3%)	p = 0.002
	Neg/*borderline* (0, +) (n=46; 74.2%)	23 (50%)	23 (50%)
ELISA for anti-fibrillarin (n=62)	Positive (O.D. ≥2.1) (n=12; 19.4%)	11 (91.7%)	1 (8.3%)	p = 0.016
	Negative (O.D. ≤2.0) (n=50; 80.6%)	27 (54%)	23 (46%)

Next, we compared the results obtained in the Fibrillarin-CBA test with IP results (**Supplementary Figure S5**) for 56 of the 62 samples with nucleolar reactivity. Of these 56 samples, 35 (62.5%) were positive and 21 (37.5%) were negative for anti-fibrillarin antibodies, with 100% agreement of positive and negative results in the Fibrillarin/CBA and IP (**Table 1**). We also analyzed the presence of anti-fibrillarin antibodies using the commercial Euroline Systemic Sclerosis Line Blot (**Supplementary Figure S6**) and found that 16 of 62 samples (25.8%) were positive and 46 (74.2%) were negative for anti-fibrillarin (**Table 1**). Of the 38 samples that were positive in the Fibrillarin/CBA test, only 15 (39.5%) were considered positive in the line blot assay (**Figure 1D**). One of the 24 samples that were negative in the Fibrillarin/CBA test was positive in the line blot assay (**Figure 1D**). Finally, we analyzed the presence of anti-fibrillarin antibody with a commercial ELISA. Out of 62 samples, 12 (19.4%) were considered positive according to the manufacturer’s recommendation for defining the cutoff (**Table 1**) and 50 (80.6%) were considered negative. Of the 38 samples with positive result in the Fibrillarin/CBA, only 11 (29%) were positive in the ELISA (**Figure 1E**). One of the 24 samples with negative result in the Fibrillarin/CBA was positive in the ELISA (**Figure 1E**).

For the Fibrillarin/CBA clinical validation, we evaluated a cohort with 106 patients classified as SSc according to the ACR/EULAR SSc classification criteria (30). The HEp-2 IFA pattern was defined according the ICAP recommendations using commercial HEp-2 slides (**Table 2**). Thirty-three patients (31.1%) presented nucleolar pattern (AC-8, AC-9, AC-10). Eleven samples (10.4% of the total) were positive in the Fibrillarin/CBA test. All these 11 samples belonged to the group of 33 samples with nucleolar pattern in the HEp-2 IFA, meaning one third (33.3%) of the samples with nucleolar pattern were positive in the Fibrillarin/CBA. None of the 73 samples with other patterns or negative in the HEp-2 IFA showed reactivity in the Fibrillarin/CBA (**Table 2**), suggesting that the CBA is very sensitive and specific for the detection of anti-fibrillarin antibodies.

**Table 2:** Anti-fibrillarin reactivity and HEp-2 IFA patterns in 106 systemic sclerosis (SSc) patients.

HEp-2 IFA pattern	Number (%) Total of 106^#^	Positive in the Fibrillarin/CBA test
Nucleolar (all) (AC-8, AC-9, AC-10)	33 (31.1%)	11 (10.4%)
Centromere (AC-3)	14 (13.2%)	0
Nuclear fine speckled (AC-4)	41 (38.7%)	0
Nuclear coarse speckled (AC-5)	6 (5.7%)	0
DNA topoisomerase I-like (AC-29)	9 (8.5%)	0
Other patterns*	13 (12.3%)	0
Negative (AC-0)	9 (8.5%)	0

*Nuclear homogeneous (AC-1), NuMA-like (AC-26), Cytoplasmic dense fine speckled (AC-19), Cytoplasmic discrete dots (AC-18), nuclear envelope (AC-11/AC-12). ^#^Some samples presented more than one staining pattern.

Demographic and clinical features of the 106 SSc patients are described in **Table 3**. SSc patients had a mean age of 50.2 ± 13.2 years, were mainly females (88.7%), with mean disease duration of 6.7 ± 5.7 years. Mean age, gender distribution and disease duration did not differ significantly between patients with positive and negative Fibrillarin/CBA results. A higher proportion of patients with positive Fibrillarin/CBA had dcSSc (72.7%), compared to patients with negative Fibrillarin/CBA test (36.8%) (p=0.022). In addition, a positive Fibrillarin/CBA was associated with a higher frequency of cardiac involvement and scleroderma renal crisis compared to those with negative Fibrillarin/CBA test (36.4% versus 6.5%, p=0.001; 18.2% versus 3.3%, p=0.028, respectively).

**Table 3:** Demographic and clinical data of systemic sclerosis (SSc) patients according to reactivity in the Fibrillarin/CBA test.

Variable	Fibrillarin/CBA positive (n = 11)	Fibrillarin/CBA negative (n = 95)	*P*
Age, mean ± SD (years)	45.2 ± 14.1	50.8 ± 13.1	0.182
Female/Male, n (%)	10 (90.9)/1 (9.1)	84 (88.4)/11 (11.6)	0.805
Disease subset, n (%) lcSSc dcSSc	3 (27.3) 8 (72.7)	60 (63.2) 35 (36.8)	0.022
Disease duration, mean ± SD (years)	5.4 ± 3.8	6.8 ± 5.9	0.652
**Organ involvement**			
Digital ulcers, n (%)	5 (45.5)	44 (47.3) (n=93)	0.907
Esophageal dysmotility, n (%)	8 (72.7)	80 (86.0) (n=93)	0.248
Small bowel involvement	2 (18.2)	9 (9.7) (n=93)	0.386
FVC % of predict, mean ± SD	72.2 ± 24.8	79.4 ± 18.9	0.306
ILD, n (%)	6 (54.5)	56 (59.6) (n=94)	0.748
PAH, n (%)	1 (9.1)	14 (15.0) (n=93)	0.595
Cardiac involvement, n (%)	4 (36.4)	6 (6.5) (n=93)	0.001
Scleroderma renal crisis, n (%)	2 (18.2)	3 (3.3) (n=93)	0.028
Arthritis or myositis, n (%)	4 (36.4)	29 (31.2) (n=93)	0.727

dcSSc, diffuse cutaneous SSc; lcSSc, limited cutaneous SSc; FVC, forced vital capacity; ILD, interstitial lung disease; PAH, pulmonary arterial hypertension.

## Discussion

SSc is a severe systemic autoimmune disease and the identification of SSc-specific autoantibodies is important not only for diagnosis but also for the prediction of clinical manifestations and outcome. In particular, the identification of anti-fibrillarin antibody is helpful because it is associated with a more severe disease and higher morbidity and mortality. In the present study we developed a new CBA for the detection of anti-fibrilarin autoantibodies that showed sensitivity and specificity compared to the gold standard IP test.

Solid-phase immunoassays (SPIA) for cell-membrane protein antigens usually present poor performance regarding sensitivity and specificity, most likely because the antigen loses its natural conformation when removed from the lipid membrane microenvironment (37, 38). Thus, for some assays we resort to animal tissue sections as substrate (37) and for others the best option is still IP with radiolabeled cell extracts. However, the latter method is not practical for routine clinical laboratories worldwide. Recent developments have demonstrated CBA technology to be an efficient strategy to replace or complement IFA on animal tissue sections and IP for detection of disease-relevant specific autoantibodies in patient samples. Application of CBA for clinical immunodiagnostics have increased in recent years, with prospect for further increase. As an example, the recent expansion in the field of neuroimmunology has largely benefited from this methodological platform as specific autoantibody biomarkers are readily determined by IFA on cells transfected with aquaporin-4, N-methyl-D-aspartate receptor (NMDAR), muscle-specific kinase (MuSK), myelin oligodendrocyte glycoprotein (MOG), among others (37). Fibrillarin is not a cell membrane protein, but suffers from a similar problem regarding the poor performance of SPIA methods. However, in contrast to the above-mentioned cell-specific autoantigens, fibrillarin is ubiquitous among eukaryotic cells.

Typically, the CBA methodology uses eukaryotic cells transfected with a vector (usually a plasmid) carrying the gene for the protein of interest. The chosen cell line should not naturally express the given autoantigen, allowing for the use of non-transfected cells as negative control. The transfection will “force” the cells to express the antigen, allowing them to be used as substrate in an indirect immunofluorescence reaction where the autoantibodies present in the patient serum are the primary probe.

In this study, we employed an innovative strategy in which the target antigen (fibrillarin) was engineered to be localized to a different cellular site from the endogenously expressed antigen. The TransMembrane Signal fused to the N-terminus of the fibrillarin transgene causes the protein to be localized to the extracellular surface of the cytoplasmic membrane, in a “receptor-like” fashion. The transfected and non-transfected cells were used as substrate in the new Fibrillarin/CBA test. This new test displayed sensitivity and specificity for detection of anti-fibrillarin antibodies equivalent to that of the gold standard IP. The high performance of the Fibrillarin/CBA test is likely due to the fact that the transgenic fibrillarin antigen, expressed on the surface of these cells, is close to its native conformation. This result encourages future studies applying a similar strategy to develop CBA tests for detection of autoantibodies to other autoantigens, especially those where traditional SPIA display suboptimal analytical and diagnostic performance.

Previous studies have shown that the nucleolar pattern may account for ~5% of all positive HEp-2 IFA results (39–42). The nucleolar pattern, especially in high titer, is also classically associated with SSc, and the proportion of nucleolar positive samples in SSc varies from 10% to 40%, depending on patient ethnicity and disease subsets evaluated (25, 26). In our study, we found that 31.1% of the SSc samples presented nucleolar pattern in the HEp-2 IFA test (some samples had additional overlapping patterns), and one third of the samples with nucleolar pattern (33.3%) were positive for anti-fibrillarin, representing 10.4% of the total SSc cohort. This frequency is similar to previous studies that have shown a prevalence of anti-fibrillarin reactivity ranging from 5 to 10% of SSc (17, 18, 26, 29). Although the mechanisms triggering high avidity autoantibodies are complex and involve a network of genetic and environmental factors, during cell death in the presence of mercury (Hg), fibrillarin seems to be altered by proteolysis, resulting in the exposure of cryptic epitopes (43), reinforcing the concept that autoantibodies are autoantigen-driven. In addition, a recent study with the relevant mice model of mercury-induced autoimmunity showed Bank1 and NF-κB as key regulators in anti-nucleolar antibody development (44). This mouse model usually produces anti-fibrillarin autoantibodies. It is not clear why several SSc patients present anti-nucleolar antibodies, but mercury-induced inflammation and autoimmunity could have a relevant contribution in susceptible people (45).

In agreement with previous studies, we found a higher frequency of diffuse cutaneous involvement (9) and higher frequency of cardiac and renal involvement (46, 47) among patients with anti-fibrillarin autoantibodies, indicating a more severe disease among these patients. However, we did not find association with GI involvement (46), ILD and PAH (17), as observed in other cohorts of anti-fibrillarin-positive SSc patients (4). The relatively small number of patients in the present cohort may have prevented the identification of association of anti-fibrillarin antibodies with these manifestations. In a study of the Pittsburgh Scleroderma Databank, evaluating 1,432 patients with SSc, anti-fibrillarin was associated with dcSSc and multi-organ involvement including joint involvement, severe gastrointestinal disease, pulmonary fibrosis, PAH, digital ulcers and heart and kidney involvement (9). Of note, a high proportion of these patients were African Americans, what could explain some of the different results observed in our patients, who had different ethnic background. Moreover, another study evaluating African American patients with SSc also found a higher frequency of digital ulcers, GI involvement, and pericarditis but less severe pulmonary involvement among patients that were anti-fibrillarin positive (18). Despite some phenotype differences among these studies, all indicate a more severe disease and decreased survival rate among patients with anti-fibrillarin antibodies (48).

Our data indicate that anti-fibrillarin antibody results based on traditional SPIA in the clinical laboratory must be taken with caution. Among the 38 Fibrillarin/CBA-positive samples, only 15 (39.5%) and 11 (29%) were considered positive in the line-blot and ELISA, respectively. The poor sensitivity of the line-blot was a surprise, especially since a previous study found that line immunoblot results for detection of anti-fibrillarin has comparable clinical significance with those of immunoprecipitation results (29). Further studies should be performed to confront or confirm these findings, including the application of new SPIA technologies such as the particle-based multi-analyte (PMAT) and fluorescence enzyme immunoassay (FEIA) (28). On the other hand, SPIA specificity was good in our study. Among the 24 samples with negative result in the Fibrillarin/CBA and IP, one was positive in the line blot assay and another one was positive in the ELISA. Although these results should be most likely false-positives, the specificity ranges around 95%.

One limitation of the fibrillarin/CBA test described in this study is the possible interference of other autoantibodies in the sample. Since the immunofluorescence pattern of TMS-fibrillarin resembles a dense fine speckled cytoplasmic pattern (**Figure 1** and **Supplementary Figure S4**), interpretation of the fibrillarin/CBA results may be skewed if a sample also contains autoantibodies against a cytoplasmic autoantigen. In this study, seven of the 106 samples of SSc patients (6.6%) gave an inconclusive result in the Fibrillarin/CBA test at initial 1/80 dilution, including four samples with dense fine speckled cytoplasmic pattern (AC-19) in standard HEp-2 IFA (none of those seven samples presented nucleolar pattern in the standard HEp-2 IFA). These seven samples were all classified as negative for anti-fibrillarin by the CBA after further testing with serial 2x dilution. Thus, in such cases, sequential double dilution can be helpful, although this double dilution process would not exclude anti-fibrillarin reactivity in samples with equivalent reactivity intensity of concomitant anti-cytoplasmic antigen.

In this proof-of-concept study, we present an innovative strategy, i.e., the overexpression of a transgenic autoantigen that was engineered to be localized to a different cellular site from the endogenously expressed antigen. This strategy overcomes one limitation in the development of CBA for autoantibodies in which the given antigen is ubiquitously expressed by the cell. The fibrillarin/CBA test described here is cost-effective and as easy to perform as a standard HEp-2 IFA reaction, making it appropriate for adoption by clinical immunodiagnostic laboratories. The performance of the Fibrillarin/CBA was comparable to the gold standard immunoprecipitation method and had higher sensitivity than commercially available anti-fibrillarin antibody solid-phase assay technologies. In addition to the successful analytical validation, Fibrillarin/CBA results were associated with disease phenotypes expected in anti-fibrillarin positive SSc, namely severe manifestations of SSc including diffuse cutaneous involvement, cardiac and renal involvement.

## Data availability statement

The original contributions presented in the study are included in the article/**Supplementary Material**. Further inquiries can be directed to the corresponding author.

## Ethics statement

The studies involving human participants were reviewed and approved by Ethics Committee of the Federal University of Sao Paulo. The patients/participants provided their written informed consent to participate in this study.

## Author contributions

GK conceived the study, designed and performed the experiments and wrote the manuscript. MS and CK critically reviewed and edited the manuscript. CK and PM collected and interpret patients’ clinical data. MS, TH, ST, LD, RA and SR handle patient samples, performed experiments and interpret data. LA conceived the study, designed the experiments, and critically reviewed and edited the manuscript. All authors have accepted responsibility for the entire content of this manuscript and approved its submission.

## Funding

This work was supported by Sao Paulo Government agency FAPESP (Sao Paulo State Research Foundation) grant numbers #2017/20745-1 and #2021/04588-9, granted to GK and LD. Additionally, LA is supported by the Brazilian research agency National Council for Research (CNPq), grant #PQ-1D 310334/2019-5. The funding organizations played no role in the study design; in the collection, analysis and interpretation of data; in the writing of the report; or in the decision to submit the report for publication.

## Conflict of interest

The authors declare that the research was conducted in the absence of any commercial or financial relationships that could be construed as a potential conflict of interest.

## Publisher’s note

All claims expressed in this article are solely those of the authors and do not necessarily represent those of their affiliated organizations, or those of the publisher, the editors and the reviewers. Any product that may be evaluated in this article, or claim that may be made by its manufacturer, is not guaranteed or endorsed by the publisher.

## References

[1] Saketkoo LA Frech T Varju C Domsic R Farrell J Gordon JK. A comprehensive framework for navigating patient care in systemic sclerosis: A global response to the need for improving the practice of diagnostic and preventive strategies in SSc. Best Pract Res Clin Rheumatol (2021) 35(3):101707. doi: 10.1016/j.berh.2021.101707 34538573 PMC8670736

[2] Roofeh D Khanna D. Management of systemic sclerosis: The first five years. Curr Opin Rheumatol (2020) 32(3):228–37. doi: 10.1097/BOR.0000000000000711 PMC9161284 32205570

[3] Damoiseaux J Andrade LEC Carballo OG Conrad K Francescantonio PLC Fritzler MJ. Clinical relevance of HEp-2 indirect immunofluorescent patterns: The international consensus on ANA patterns (ICAP) perspective. Ann rheum Dis (2019) 78(7):879–89. doi: 10.1136/annrheumdis-2018-214436 PMC6585284 30862649

[4] Mehra S Walker J Patterson K Fritzler MJ. Autoantibodies in systemic sclerosis. Autoimmun Rev (2013) 12(3):340–54. doi: 10.1016/j.autrev.2012.05.011 22743034

[5] Walker UA Tyndall A Czirjak L Denton C Farge-Bancel D Kowal-Bielecka O. Clinical risk assessment of organ manifestations in systemic sclerosis: A report from the EULAR scleroderma trials and research group database. Ann rheum Dis (2007) 66(6):754–63. doi: 10.1136/ard.2006.062901 PMC1954657 17234652

[6] Shah AA Hummers LK Casciola-Rosen L Visvanathan K Rosen A Wigley FM. Examination of autoantibody status and clinical features associated with cancer risk and cancer-associated scleroderma. Arthritis Rheumatol (2015) 67(4):1053–61. doi: 10.1002/art.39022 PMC4400658 25605296

[7] Kuwana M Kaburaki J Okano Y Tojo T Homma M. Clinical and prognostic associations based on serum antinuclear antibodies in Japanese patients with systemic sclerosis. Arthritis rheum (1994) 37(1):75–83. doi: 10.1002/art.1780370111 8129766

[8] Hu PQ Fertig N Medsger TA Jr. Wright TM. Correlation of serum anti-DNA topoisomerase I antibody levels with disease severity and activity in systemic sclerosis. Arthritis rheum (2003) 48(5):1363–73. doi: 10.1002/art.10977 12746909

[9] Steen VD. Autoantibodies in systemic sclerosis. Semin Arthritis rheum (2005) 35(1):35–42. doi: 10.1016/j.semarthrit.2005.03.005 16084222

[10] Lazzaroni MG Cavazzana I Colombo E Dobrota R Hernandez J Hesselstrand R. Malignancies in patients with anti-RNA polymerase III antibodies and systemic sclerosis: Analysis of the EULAR scleroderma trials and research cohort and possible recommendations for screening. J Rheumatol (2017) 44(5):639–47. doi: 10.3899/jrheum.160817 28089973

[11] Ceribelli A Cavazzana I Airo P Franceschini F. Anti-RNA polymerase III antibodies as a risk marker for early gastric antral vascular ectasia (GAVE) in systemic sclerosis. J Rheumatol (2010) 37(7):1544. doi: 10.3899/jrheum.100124 20595295

[12] Hamaguchi Y Kodera M Matsushita T Hasegawa M Inaba Y Usuda T. Clinical and immunologic predictors of scleroderma renal crisis in Japanese systemic sclerosis patients with anti-RNA polymerase III autoantibodies. Arthritis Rheumatol (2015) 67(4):1045–52. doi: 10.1002/art.38994 25512203

[13] Patterson KA Roberts-Thomson PJ Lester S Tan JA Hakendorf P Rischmueller M. Interpretation of an extended autoantibody profile in a well-characterized Australian systemic sclerosis (Scleroderma) cohort using principal components analysis. Arthritis Rheumatol (2015) 67(12):3234–44. doi: 10.1002/art.39316 26246178

[14] Iyer-Bierhoff A Krogh N Tessarz P Ruppert T Nielsen H Grummt I. SIRT7-dependent deacetylation of fibrillarin controls histone H2A methylation and rRNA synthesis during the cell cycle. Cell Rep (2018) 25(11):2946–54 e5. doi: 10.1016/j.celrep.2018.11.051 30540930

[15] Yip WS Shigematsu H Taylor DW Baserga SJ. Box C/D sRNA stem ends act as stabilizing anchors for box C/D di-sRNPs. Nucleic Acids Res (2016) 44(18):8976–89. doi: 10.1093/nar/gkw576 PMC5062973 27342279

[16] Omer AD Ziesche S Ebhardt H Dennis PP. *In vitro* reconstitution and activity of a C/D box methylation guide ribonucleoprotein complex. Proc Natl Acad Sci United States America (2002) 99(8):5289–94. doi: 10.1073/pnas.082101999 PMC122762 11959980

[17] Aggarwal R Lucas M Fertig N Oddis CV Medsger TA Jr. Anti-U3 RNP autoantibodies in systemic sclerosis. Arthritis rheum (2009) 60(4):1112–8. doi: 10.1002/art.24409 19333934

[18] Sharif R Fritzler MJ Mayes MD Gonzalez EB McNearney TA Draeger H. Anti-fibrillarin antibody in African American patients with systemic sclerosis: Immunogenetics, clinical features, and survival analysis. J Rheumatol (2011) 38(8):1622–30. doi: 10.3899/jrheum.110071 PMC3149738 21572159

[19] Kayser C Fritzler MJ. Autoantibodies in systemic sclerosis: Unanswered questions. Front Immunol (2015) 6:167. doi: 10.3389/fimmu.2015.00167 25926833 PMC4397862

[20] Chan EKL von Muhlen CA Fritzler MJ Damoiseaux J Infantino M Klotz W. The international consensus on ANA patterns (ICAP) in 2021-the 6th workshop and current perspectives. J Appl Lab Med (2022) 7(1):322–30. doi: 10.1093/jalm/jfab140 34996073

[21] Reveille JD Fischbach M McNearney T Friedman AW Aguilar MB Lisse J. Systemic sclerosis in 3 US ethnic groups: A comparison of clinical, sociodemographic, serologic, and immunogenetic determinants. Semin Arthritis rheum (2001) 30(5):332–46. doi: 10.1053/sarh.2001.20268 11303306

[22] Ferri C Bernini L Cecchetti R Latorraca A Marotta G Pasero G. Cutaneous and serologic subsets of systemic sclerosis. J Rheumatol (1991) 18(12):1826–32. 1795320

[23] Bunn CC Denton CP Shi-Wen X Knight C Black CM. Anti-RNA polymerases and other autoantibody specificities in systemic sclerosis. Br J Rheumatol (1998) 37(1):15–20. doi: 10.1093/rheumatology/37.1.15 9487245

[24] Hesselstrand R Scheja A Shen GQ Wiik A Akesson A. The association of antinuclear antibodies with organ involvement and survival in systemic sclerosis. Rheumatology (2003) 42(4):534–40. doi: 10.1093/rheumatology/keg170 12649400

[25] Nandiwada SL Peterson LK Mayes MD Jaskowski TD Malmberg E Assassi S. Ethnic differences in autoantibody diversity and hierarchy: More clues from a US cohort of patients with systemic sclerosis. J Rheumatol (2016) 43(10):1816–24. doi: 10.3899/jrheum.160106 27481902

[26] Satoh M Ceribelli A Hasegawa T Tanaka S. Clinical significance of antinucleolar antibodies: Biomarkers for autoimmune diseases, malignancies, and others. Clin Rev Allergy Immunol (2022) 63(2):210–39. doi: 10.1007/s12016-022-08931-3 35258843

[27] Stochmal A Czuwara J Trojanowska M Rudnicka L. Antinuclear antibodies in systemic sclerosis: an update. Clin Rev Allergy Immunol (2020) 58(1):40–51. doi: 10.1007/s12016-018-8718-8 30607749

[28] Mahler M Kim G Roup F Bentow C Fabien N Goncalves D. Evaluation of a novel particle-based multi-analyte technology for the detection of anti-fibrillarin antibodies. Immunol Res (2021) 69(3):239–48. doi: 10.1007/s12026-021-09197-1 PMC8266783 33913080

[29] Peterson LK Jaskowski TD Mayes MD Tebo AE. Detection of anti-U3-RNP/fibrillarin IgG antibodies by line immunoblot assay has comparable clinical significance to immunoprecipitation testing in systemic sclerosis. Immunol Res (2016) 64(2):483–8. doi: 10.1007/s12026-015-8710-9 26467972

[30] van den Hoogen F Khanna D Fransen J Johnson SR Baron M Tyndall A. 2013 classification criteria for systemic sclerosis: an American college of rheumatology/European league against rheumatism collaborative initiative. Ann rheum Dis (2013) 72(11):1747–55. doi: 10.1136/annrheumdis-2013-204424 24092682

[31] de Oliveira SM Martins LVO Lupino-Assad AP Medeiros-Ribeiro AC de Moraes DA Del-Rio APT. Severity and mortality of COVID-19 in patients with systemic sclerosis: A Brazilian multicenter study. Semin Arthritis rheum (2022) 55:151987. doi: 10.1016/j.semarthrit.2022.151987 35286906 PMC8875950

[32] Keppeke GD Prado MS Nunes E Perazzio SF Rodrigues SH Ferraz ML. Differential capacity of therapeutic drugs to induce Rods/Rings structures *in vitro* and *in vivo* and generation of anti-Rods/Rings autoantibodies. Clin Immunol (2016) 173:149–56. doi: 10.1016/j.clim.2016.10.004 27746381

[33] Reimer G Pollard KM Penning CA Ochs RL Lischwe MA Busch H. Monoclonal autoantibody from a (New Zealand black x new Zealand white)F1 mouse and some human scleroderma sera target an Mr 34,000 nucleolar protein of the U3 RNP particle. Arthritis rheum (1987) 30(7):793–800. doi: 10.1002/art.1780300709 2441711

[34] Keppeke GD Satoh M Ferraz ML Chan EK Andrade LE. Temporal evolution of human autoantibody response to cytoplasmic rods and rings structure during anti-HCV therapy with ribavirin and interferon-alpha. Immunol Res (2014) 60(1):38–49. doi: 10.1007/s12026-014-8515-2 24845459

[35] Carcamo WC Ceribelli A Calise SJ Krueger C Liu C Daves M. Differential reactivity to IMPDH2 by anti-rods/rings autoantibodies and unresponsiveness to pegylated interferon-alpha/ribavirin therapy in US and Italian HCV patients. J Clin Immunol (2013) 33(2):420–6. doi: 10.1007/s10875-012-9827-4 23100146

[36] Satoh M Langdon JJ Hamilton KJ Richards HB Panka D Eisenberg RA. Distinctive immune response patterns of human and murine autoimmune sera to U1 small nuclear ribonucleoprotein c protein. J Clin Invest (1996) 97(11):2619–26. doi: 10.1172/JCI118711 PMC507349 8647956

[37] Molina RD Conzatti LP da Silva APB Goi LDS da Costa BK Machado DC. Detection of autoantibodies in central nervous system inflammatory disorders: Clinical application of cell-based assays. Mult scler relat Disord (2020) 38:101858. doi: 10.1016/j.msard.2019.101858 31775115

[38] Oger J Frykman H. An update on laboratory diagnosis in myasthenia gravis. Clin chim acta Int J Clin Chem (2015) 449:43–8. doi: 10.1016/j.cca.2015.07.030 26238187

[39] Satoh M Chan EK Ho LA Rose KM Parks CG Cohn RD. Prevalence and sociodemographic correlates of antinuclear antibodies in the united states. Arthritis rheum (2012) 64(7):2319–27. doi: 10.1002/art.34380 PMC3330150 22237992

[40] Dinse GE Parks CG Weinberg CR Co CA Wilkerson J Zeldin DC. Increasing prevalence of antinuclear antibodies in the united states. Arthritis Rheumatol (2020) 72(6):1026–35. doi: 10.1002/art.41214 PMC7255943 32266792

[41] Sener AG Afsar I Demirci M. Evaluation of antinuclear antibodies by indirect immunofluorescence and line immunoassay methods': Four years' data from Turkey. APMIS Acta pathol microbiol immunol Scand (2014) 122(12):1167–70. doi: 10.1111/apm.12275 24735346

[42] Mengeloglu Z Tas T Kocoglu E Aktas G Karabork S. Determination of anti-nuclear antibody pattern distribution and clinical relationship. Pakistan J Med Sci (2014) 30(2):380–3. doi: 10.12669/pjms.302.4276 PMC3999014 24772147

[43] Pollard KM Lee DK Casiano CA Bluthner M Johnston MM Tan EM. The autoimmunity-inducing xenobiotic mercury interacts with the autoantigen fibrillarin and modifies its molecular and antigenic properties. J Immunol (1997) 158(7):3521–8. 9120314

[44] Alkaissi H Havarinasab S Nielsen JB Soderkvist P Hultman P. Bank1 and NF-kappaB as key regulators in anti-nucleolar antibody development. PloS One (2018) 13(7):e0199979. doi: 10.1371/journal.pone.0199979 30016332 PMC6049909

[45] Pollard KM Cauvi DM Toomey CB Hultman P Kono DH. Mercury-induced inflammation and autoimmunity. Biochim Biophys Acta Gen Subj (2019) 1863(12):129299. doi: 10.1016/j.bbagen.2019.02.001 30742953 PMC6689266

[46] Arnett FC Reveille JD Goldstein R Pollard KM Leaird K Smith EA. Autoantibodies to fibrillarin in systemic sclerosis (scleroderma). An immunogenetic, serologic, and clinical analysis. Arthritis Rheum (1996) 39(7):1151–60. doi: 10.1002/art.1780390712 8670324

[47] Tormey VJ Bunn CC Denton CP Black CM. Anti-fibrillarin antibodies in systemic sclerosis. Rheumatology (2001) 40(10):1157–62. doi: 10.1093/rheumatology/40.10.1157 11600746

[48] Kuwana M. Circulating anti-nuclear antibodies in systemic sclerosis: Utility in diagnosis and disease subsetting. J Nippon Med Sch = Nippon Ika Daigaku zasshi (2017) 84(2):56–63. doi: 10.1272/jnms.84.56 28502960

