# Supplementary material for: A cell-based assay for detection of anti-fibrillarin autoantibodies with performance equivalent to immunoprecipitation
Source: Front Immunol. 2022 Sep 26;13:1011110. doi: 10.3389/fimmu.2022.1011110 (PMC9549361; doi:10.3389/fimmu.2022.1011110)
Supplement: Supplementary file 1 [file DataSheet_1.pdf]

A cell-based assay for detection of anti-fibrillarin autoantibodies with performance equivalent to immunoprecipitation

Supplementary Material

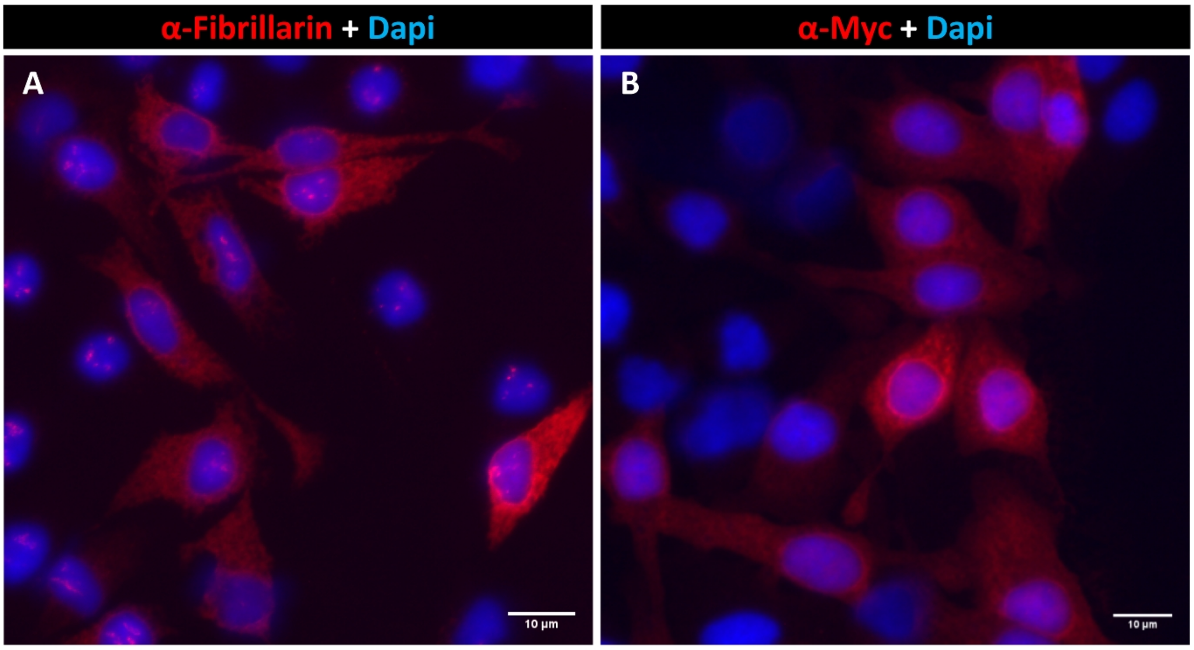

Suppl. Figure 1. TMS-fibrillarin is localized to the cell membrane. HEp-2 cells transfected with the pCMV_TMS-fibrillarin_P2A_OFP-myc plasmid were probed with mouse monoclonal antibodies and counterstained with DAPI to visualize nuclear DNA. (A) Anti-fibrillarin labeling. (B) Anti-myc labeling. Scale bar = 10μm.

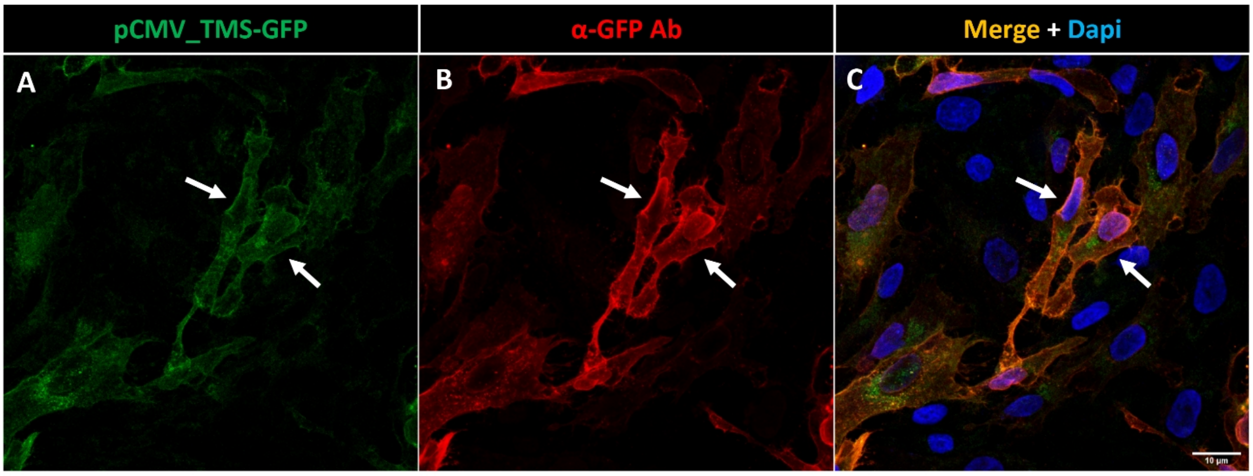

Suppl. Figure 2. TMS-GFP is localized to the cell membrane. (A-B) HeLa cells overexpressing TMS-GFP (green) were probed with anti-GFP antibody (red). (C) Merge plus DNA counterstaining with DAPI (blue). Arrows indicate cells expressing the TMS-GFP labeled by the anti-GFP antibody. Scale bar = 10μm.

Detailed methods for this experiment: HeLa cells were transfected with a pCMV_TMS-GFP plasmid. The plasmid was constructed by fusing the TMS signal to the N' terminus of GFP (pCMV_GFP backbone plasmid from Sino Biological, China). Twenty-four hours later cells were fixed with 4% paraformaldehyde and probed with a mouse monoclonal anti-GFP antibody (11814460001, Roche, Switzerland), followed by an anti-mouse IgG conjugated to Cy3 (715-165-151, Jackson ImmunoResearch, USA). To preserve cell membrane stability, the detergent-based permeabilization step was omitted. Fluorescent images were acquired with a laser-scanning confocal microscope (TCS SP8 STED 3X, Leica, Germany).

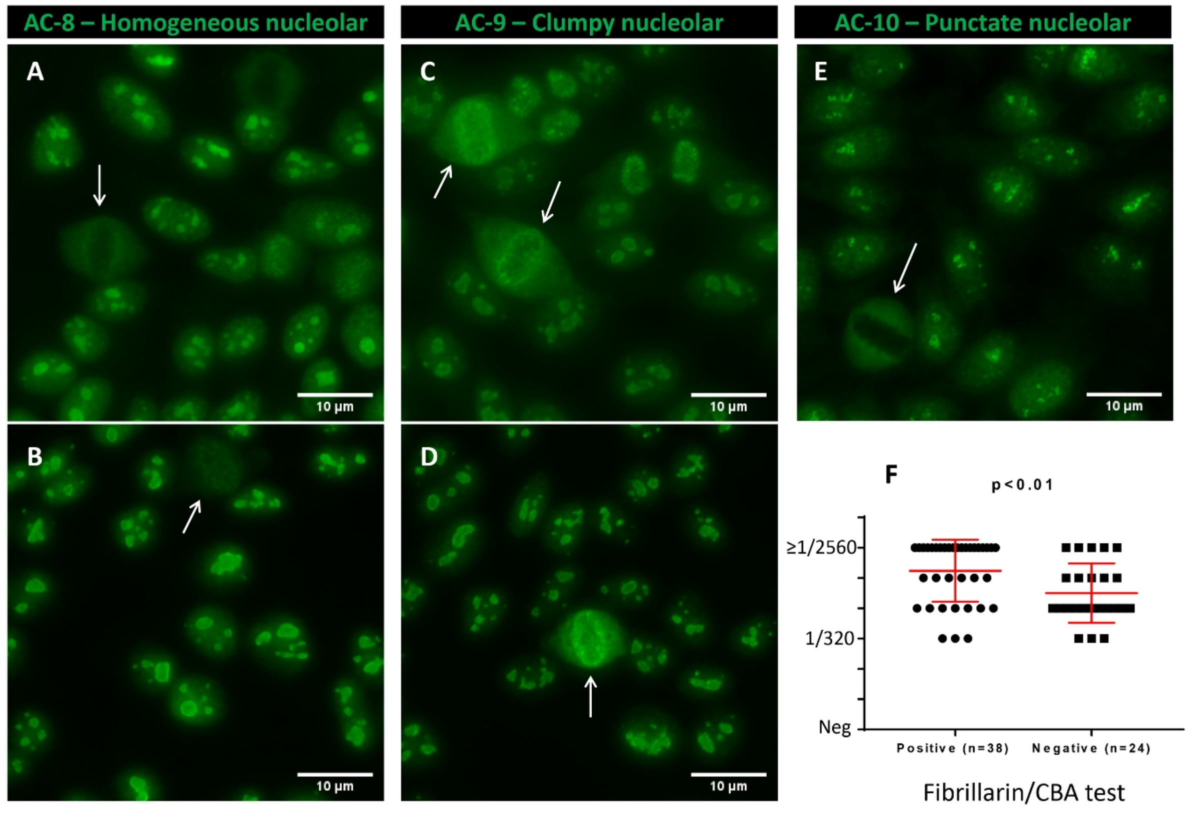

Suppl. Figure 3. Different nucleolar patterns in the HEp-2 IFA test. Example of samples presenting homogenous nucleolar AC-8 (A – B), clumpy nucleolar AC-9 (C – D) and punctate nucleolar AC-10 (E). Peri-chromosomal staining at the metaphase plates is frequently observed in the clumpy nucleolar pattern. Arrows in all panels indicate metaphase plates. Scale bar = 10μm. (F) Distribution of HEp-2 IFA titer in samples positive or negative for anti-fibrillarin in the Fibrillarin/CBA test. Error bars indicate mean ± S.D.

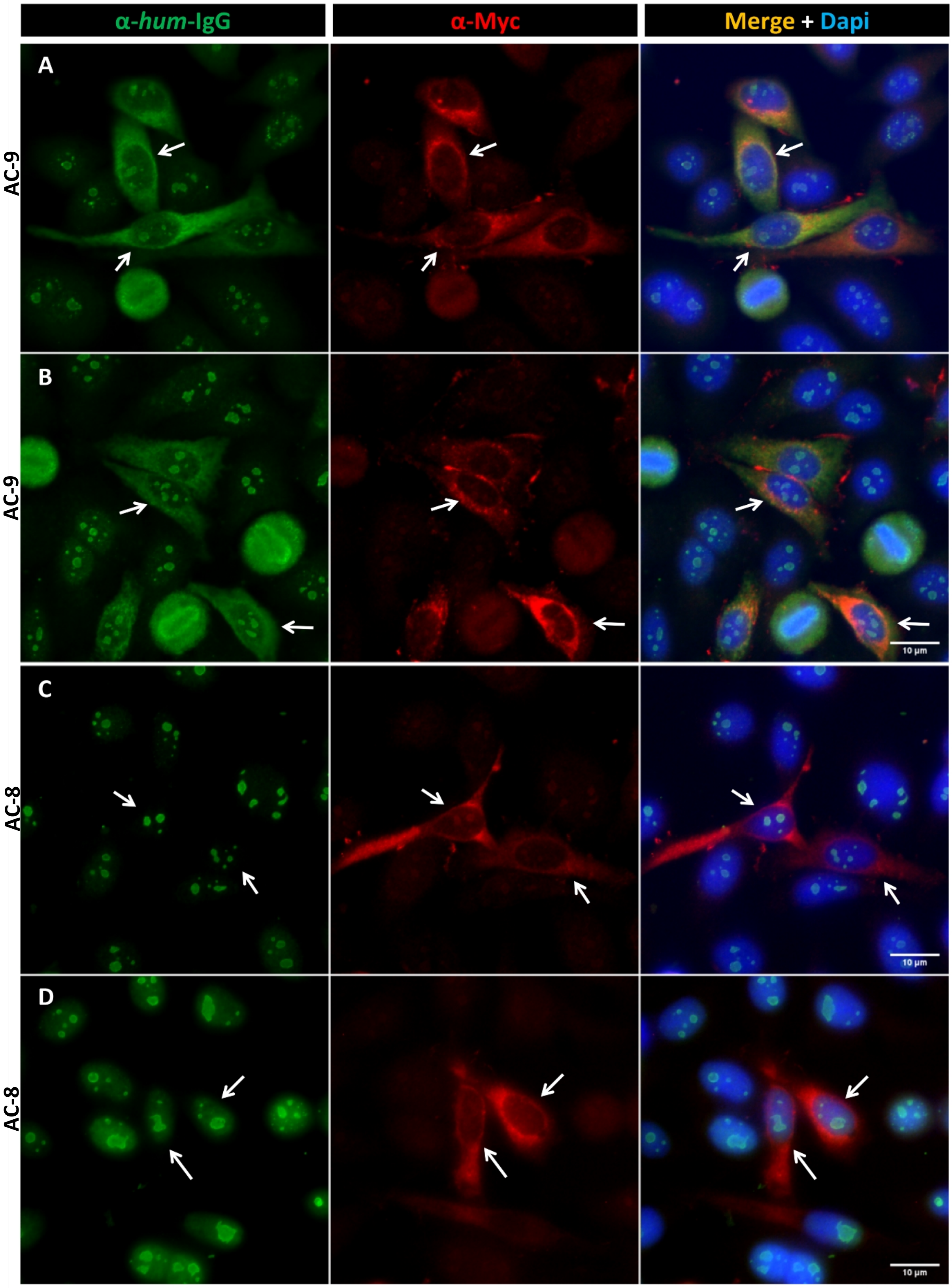

Suppl. Figure 4. Examples of positive and negative results in the Fibrillarin/CBA test. (A-B) Examples of positive samples. (C-D) Examples of negative samples. Serum samples were used at 1/80 dilution. Arrows indicate cells expressing TMS-fibrillarin. Scale bar = 10µm.

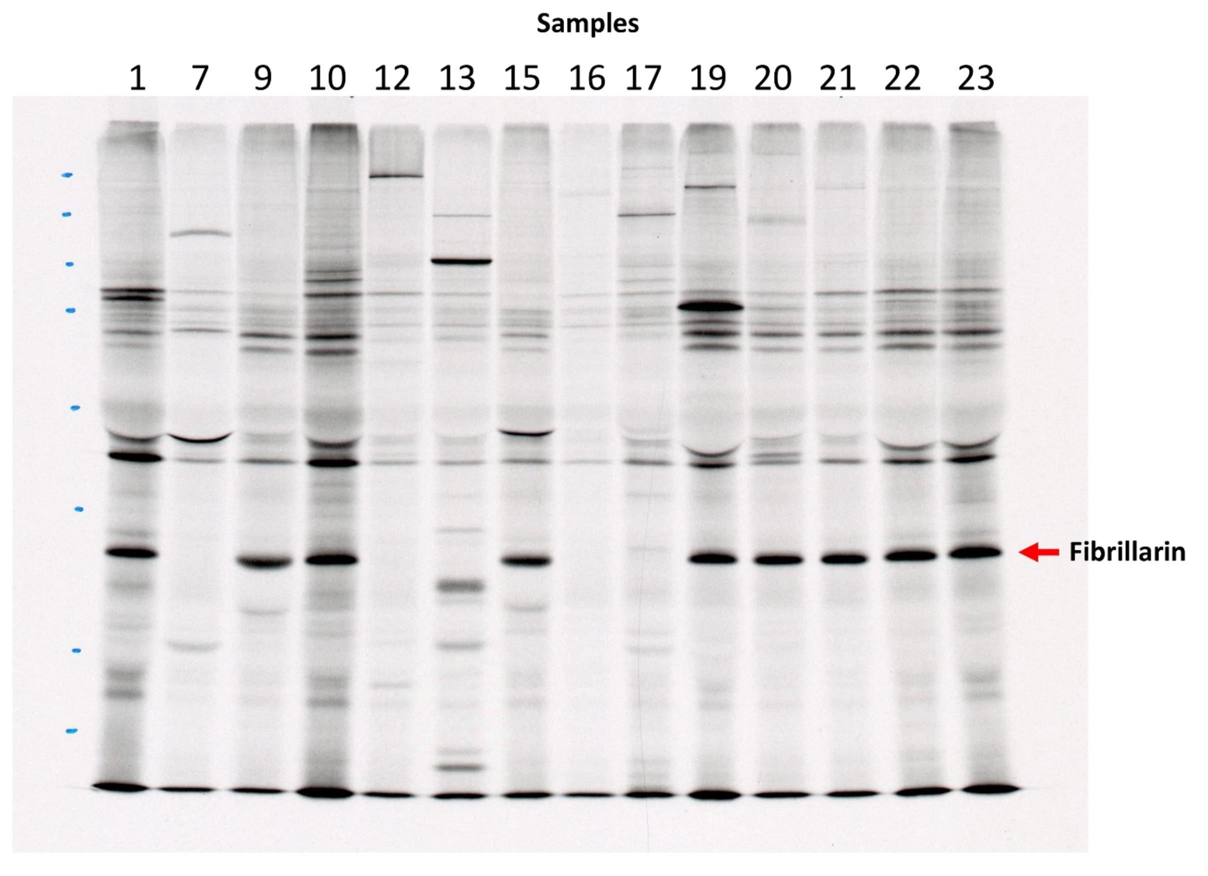

Suppl. Figure 5. Immunoprecipitation for detection of reactivity to fibrillarin. Representative gel showing reactivity with the ~34kDa fibrillarin band (red arrow) with nine samples and no reactivity with five samples.

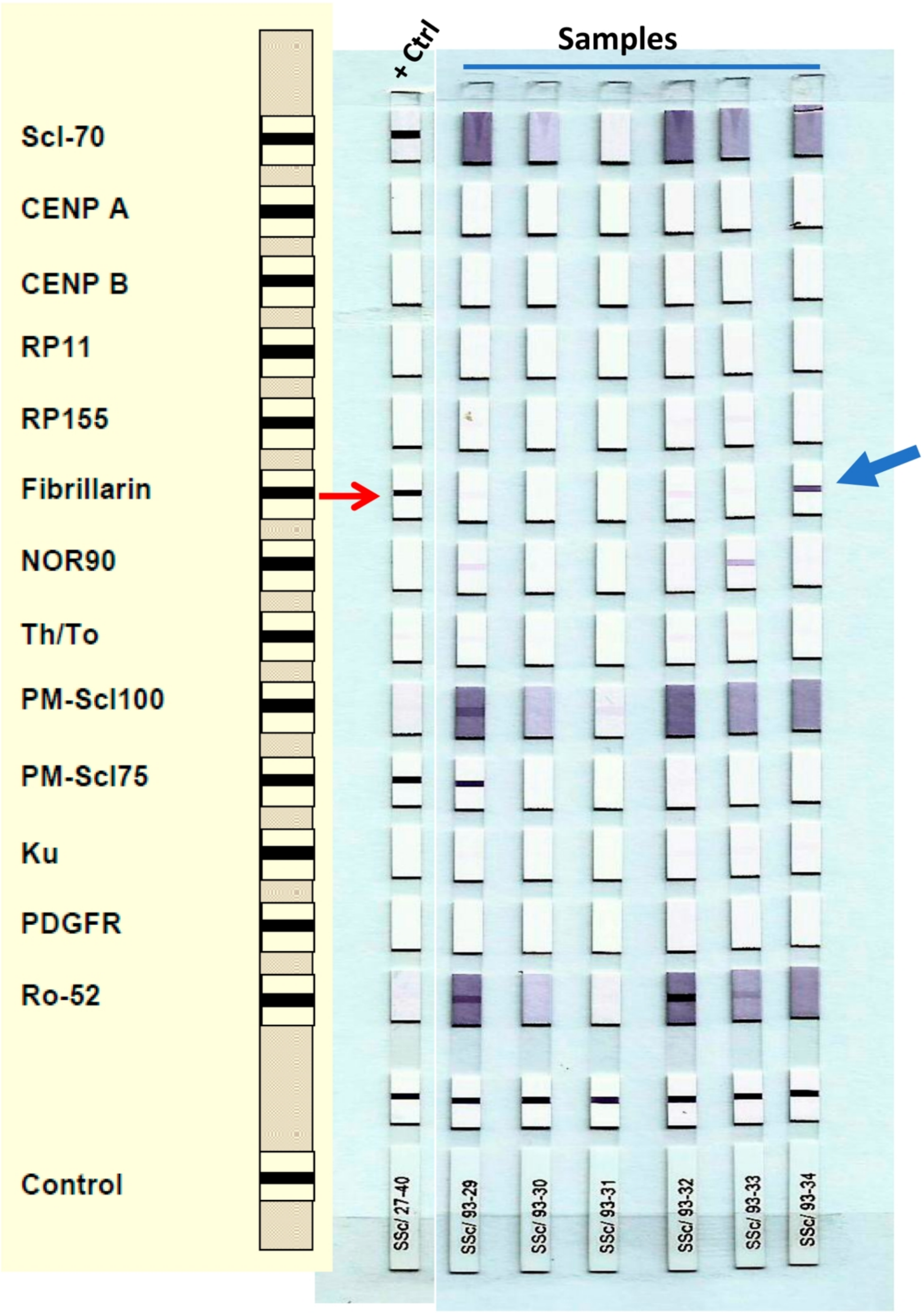

Suppl. Figure 6. Representative line blot assay. For the line blot analyses, Euroline Systemic sclerosis (Nucleoli) profile was used. Red arrow indicates the fibrillarin lane. Blue arrow indicates a positive sample.
